# Target Type Modulates the Effect of Task Demand on Reflexive Focal Attention

**DOI:** 10.3390/vision1020013

**Published:** 2017-05-06

**Authors:** Andrea Albonico, Manuela Malaspina, Roberta Daini

**Affiliations:** 1 Human Vision and Eye Movement Laboratory, Departments of Medicine (Neurology), Ophthalmology and Visual Sciences, University of British Columbia, Vancouver, BC V5Z 3N9 Canada; 2 NeuroMI—Milan Center for Neuroscience, Milano 20126, Italy; 3 Psychology Department, Università degli Studi di Milano-Bicocca, Milano 20126, Italy; 4 COMiB—Optics and Optometry Research Center, Università degli Studi di Milano-Bicocca, Milano 20126, Italy

**Keywords:** focal attention, spatial attention, central vision, reflexive attention, detection, discrimination

## Abstract

Focusing attention on a limited space within the environment allows us to concentrate our resources selectively on that location while ignoring the rest of the space. In this study we investigated how the deployment of the focal attention in foveal vision can be affected by task and stimuli specificity. In particular, we measured the cue-size effect in four experiments: shape detection (Experiment 1), shape discrimination (Experiment 2), letter detection (Experiment 3), and letter discrimination (Experiment 4). Our results highlight that, although the focal component can be elicited by different tasks (i.e., detection or discrimination) and by using different types of stimuli (i.e., shapes or letters), those effects interact with each other. Specifically, the effect of focal attention is more noticeable when letter stimuli are used in the case of a detection task, while no difference between letters and geometrical shapes is observed in the discrimination task. Furthermore, the analysis of the cue-size effect across the four experiments confirmed that the deployment of focal attention in foveal vision is mainly reflexive.

## 1. Introduction

Spatial attention can be defined as the mechanism that addresses the processing resources onto the external sensory input by its spatial location and withdraws processing resources from the many competing stimuli present in the environment [1]. Whereas a lot of studies have investigated how attentional resources are shifted from one location to another in the environment (here referred as orienting attention), considerably fewer studies have explored how attentional resources can be selectively concentrated on a limited ‘spatial window’ within the environment while ignoring the rest of it (here referred to as focal attention).

Focal attention consists of the ability to adjust the size and shape of the focus of attention, with a subsequent increase of the efficiency of the processing of specific locations or objects within it [2]. Focal attention is a distinct and independent mechanism of orienting [3,4]. In particular, Albonico, Malaspina, Bricolo, Martelli and Daini [4] described focal attention as a reflexive component that contributes to spatial resolution in foveal vision. Different studies have demonstrated the existence of an inverse relation between the size of the focus of attention, induced by a cue, and the efficiency in the processing within it, i.e., the cue-size effect [5,6,7,8]. Converging results come also from some neuroimaging studies [9,10], demonstrating that the level of neural activity in the visual cortex is inversely correlated with the size of the attended region. Despite contrasting results in the literature regarding its temporal characteristics [5,6,7,8,11], it has been demonstrated that focal attention affects different aspects of visual cognition; most of the studies have focused on the speed and accuracy with which a target stimulus can be detected or discriminated [5,6,7,8,12], but focal attention can also affect contrast sensitivity [13] and visual sensory memory [14].

Thus, although focal attention has been the topic of several studies, still little is known about the many factors that might affect the cue-size effect. Of particular importance it is how the task demands and specific stimuli used can influence the deployment of focal attention. Similar issues have been already addressed in the domain of orienting. Indeed, some studies [15,16,17] showed that orienting can be modulated differently between detection and discrimination tasks and that the use of different stimuli can affect orienting as well [18,19,20]. For instance, previous studies have shown that biologically relevant stimuli elicit orienting in a way that is qualitatively different compared to nonbiologically relevant stimuli [21,22].

The studies investigating focal attention mentioned above made use of different paradigms, and most of them did not take into account the effect of task demands. In particular, whereas some studies used detection tasks [8,11], others used discrimination tasks [5,23,24], and it could be possible that the characteristics of the cue-size effect can be revealed differently by using different task paradigms. In fact, it has been argued that discrimination tasks rely on attention more than detection tasks do [25,26], and this difference may be due to the fact that detection requires the visual system to solve a simpler problem than discrimination [5,23,24,27,28]. Thus, according to some authors, whilea simple detection task is not suitable for studying the effects of the focal component because of its lack of sensitivity to the degree of concentration of attentional resources, a discrimination task would be best-suited because it requires more attentional resources [5,23,24,27,28]. On the other side, some authors [7,11,29,30] have argued that the use of recognition or discrimination tasks is not really optimal to study the processing of focusing attention, given that, in addition to visual attention, other processes like expectation (categorization of the stimulus) and intention (selection of the correct response) are involved during these tasks [30,31]. According to these authors, the use of a simple detection task is perfectly suitable for studying focal attention, because even the detection of simple features is not possible without focal attention [32]. Thus, to date, it still has to be clarified whether the task demands have an effect on the deployment of the focal component or if the characteristics and the temporal dynamics of this component can be disclosed by both detection and discrimination tasks. To our knowledge, only Turatto, Benso, Facoetti, Galfano, Mascetti and Umiltà [3] used both a simple detection and a discrimination task, providing first evidence that the cue-size effect can be obtained with both tasks. However, even in this case, a direct comparison of the cue-size effects obtained with the two paradigms was missing.

Furthermore, whereas in the orienting domain some studies have suggested that the type of stimuli used or the different perception we have of those stimuli can affect the orienting of visual attention [18,19,20,33,34,35,36,37], the effect of the stimulus used has not been investigated directly in the domain of the focal attention, and a clear answer to this question cannot be given yet. In particular, in this study, we focused on the comparison between letters and geometrical shapes. Letters were used because of their ‘special’ status. Indeed, letters not only play a critical role in reading [38] but different studies have also demonstrated that they have specific properties: indeed, letters are characterized by automatic processing [39,40], and efficiency in processing letter information accurately is acquired very early in life [41]. Moreover, attention to letters has a finer spatial extent than other types of stimuli [24,42]. Furthermore, letters are also widely used as target stimuli in multiple paradigms in order to investigate other aspects of the human visual system such as crowding [43] and visual search [44]. Thus, in order to have a better understanding of the factors that influence the deployment of attention, it is critical to investigate whether the use of different paradigms (detection vs. discrimination tasks) leads to different results when letter stimuli are used. As a comparison, geometrical shapes were chosen because of their wide use in the focal attention literature [3,8,11,45]. Moreover, because their basic components (straight or curved lines) are similar to the ones that constitute letters, geometrical shapes represent comparable control stimuli for letters.

Therefore, the aim of the present study was to investigate in detail how the deployment of focal attention could be affected by different task demands and different stimuli. In particular, we believe that a better understanding of how different tasks and stimuli affect how the cue-size effect can be evoked is critical, not only to reconcile the discrepancies across different studies but also to obtain a deeper comprehension of focal attention. The importance of a better understading of this function is also supported by the fact that some studies have suggested that focal attention could play an important role in normal reading [46,47], developmental dyslexia [48,49], and also in the cognitive dysfunction related to normal ageing [50,51]. Thus, enhancing our knowledge of focal attention would be important also to increase our understanding of significant topics such as normal and pathological reading and ageing. To this aim, we performed a series of experiments to compare the effects of task demand and stimulus type on focal attention at different stimulus onset asynchronies (SOAs). In particular, similarly to Maringelli and Umiltà [8], different SOAs were used in the experiments in order to study the time course of focusing and, thus, to further investigate whether focusing is an automatically triggered mechanism or a voluntary mechanism under top-down control. For this reason, here we carried out four different experiments in which the task and the target stimulus were, respectively: shape detection (Experiment 1), shape discrimination (Experiment 2), letter detection (Experiment 3), and letter discrimination (Experiment 4).

## 2. Experiment 1: Shape Detection

In this first experiment, we used the same paradigm developed by Maringelli and Umiltà [8], with a red dot acting as the target stimulus and two possible squares of different sizes as cues. The order of presentation of the cues of different sizes was manipulated, so that the cue size was fixed in one block and randomized in another. Three possible stimulus onset asynchronies (SOAs) allowed us to follow the time course of focal attention. The aim of Experiment 1 was to replicate the original results by Maringelli and Umiltà [8] and, thus, to demonstrate the existence of a significant cue-size effect only when the order of presentation of the cue was randomized and at shorter SOAs. In particular, according to Maringelli and Umiltà [8], the lack of attentional effects in the fixed sequence would suggest that, even if mainly driven by reflexive mechanisms, the attentional focus could still be modulated by endogenous factors.

### 2.1. Materials and Methods

#### 2.1.1. Participants

Twenty healthy volunteers (12 females, mean age = 24.16 ± 1.38, range = 22–27) took part in Experiment 1. All participants had normal or corrected-to-normal vision and were right-handed. None of them had neurological, psychiatric, or other relevant medical problems.

Participants were all naïve to the experimental procedure and to the purpose of the study. They gave informed consent prior to being enrolled in the study, which was carried out according to the guidelines of the ethical committee of the University of Milano-Bicocca (Prot. #RM-2016-32) and in accordance with the ethical standards of the Declaration of Helsinki.

#### 2.1.2. Apparatus, Stimuli and Procedure

Participants were seated in front of a computer monitor (19-in, 400 × 300 mm) with a resolution of 1280 × 1024 pixels and a refresh rate of 60 Hz. A chin and forehead rest stabilized their head position and kept the viewing distance constant at 57 cm. Stimulus presentation, timing and response recording were carried out by the E-prime Software (Psychology Software Tools, Pittsburgh, PA, USA).

The cue stimulus consisted of an empty square of variable size (3° × 3° or 6° × 6°, thickness of 0.1°), outlined in white on a black background. The target stimulus was a red dot (diameter of 0.4°). The cue stimulus was always presented in the centre of the screen and the target stimulus was always shown in the centre of the cue. A response to the target stimulus was made by pressing the space-bar on the computer keyboard.

**Figure 1:**
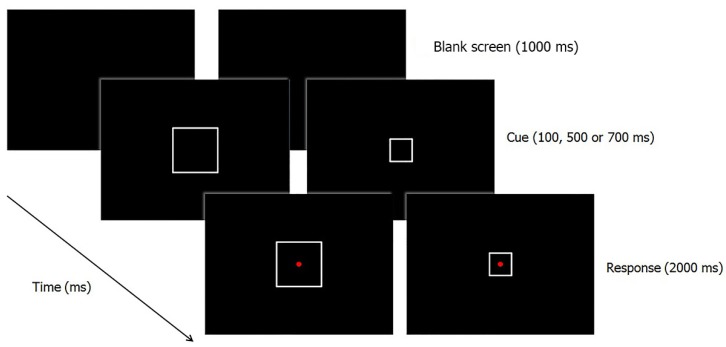
Example of a trial procedure in Experiment 1. After a blank screen, the target appearance could be preceded by a small cue (on the right), or a big cue (on the left). After 100, 500, or 700 ms the target stimulus appeared and the participant was asked to respond as quickly as possible by pressing the space bar.

Each trial started with a blank black screen, followed 1000 ms later by one of the two possible cues (we chose not to display any fixation point to avoid giving an additional or confounding cue to participants). The cue remained on the screen until the end of the trial, while the target stimulus appeared after one of these possible SOAs: 100, 500, or 700 ms. The target stimulus remained on the screen until the participant responded (in any case for a maximum of 2000 ms), after which the target disappeared and the next trial started (see Figure 1). On 20% of trials no target stimulus was presented (i.e., catch trials). Participants were instructed to fixate on the centre of the screen, to focus attention inside the square, and to press the space-bar as quickly as possible in response to the target stimulus. They were also instructed to refrain from responding to catch trials.

Every participant completed 240 trials, divided into two blocks of 120 trials each; one block in which the cues were presented randomly (random block) and a second one in which the order of presentation of the cues was fixed (fixed block). In this latter condition, the order of the presentation of the cues followed an ABBA scheme in which A consisted of 30 trials with the small cue and B of 30 trials with the big cue. Half of the participants started the experiment with the random block, while the remaining half started with the fixed block. The trials were also equally divided between the 100, 500, and 700 ms SOA conditions, as well as between the two conditions of the cue (for a total of 16 trials for a single condition).

At the beginning of the experiment, a practice session composed of 10 trials (equally divided for the cueing condition and randomly selected regarding the SOA condition) was run in order to let the participants familiarize themselves with the task and to practice with response modality.

#### 2.1.3. Statistical Analyses

Statistical analyses were performed using the software IBM SPSS Statistics 22 (IBM Corp, Armonk, NY, USA). Reaction times (RTs, measured from the onset of the target stimulus to response emission) were analysed via a repeated-measures Analysis of Variance (ANOVA). Significant differences were further explored by Bonferroni post-hoc multiple comparisons, and corrected *p*-values are reported. The effect size in the ANOVA was also measured by computing the Eta Squared (η^2^), which expresses the degree of association between an effect and the dependent variable, i.e., the proportion of the total variance that is attributable to a factor [52]. Trials with false alarms, i.e., responses to catch trials, and atypical RTs outliers (employing as a criterion 2.5 standard deviations, (SDs), above or below the mean within each participant) were discarded and not analysed (2.6% of trials were discarded). Results from the random and fixed blocks were analysed separately.

The cue-size effect was also computed in order to obtain an amodal quantification of the advantage of narrowing the focal component comparable across the four experiments. To this aim, using the same procedure as the one used in Albonico, Malaspina, Bricolo, Martelli and Daini [4], Cohen’s *d* between the big square and the small square conditions was computed. This measure was used because, compared to the simple subtraction of the mean RTs for the small cue from the mean RTs for the big cue, it also takes into account the variability in the response speed.

### 2.2. Results

On catch trials, participants committed very few false alarms (less than 1% in the fixed block and none in the random block), with no differences among conditions; thus these data were not further analysed.

**Figure 2:**
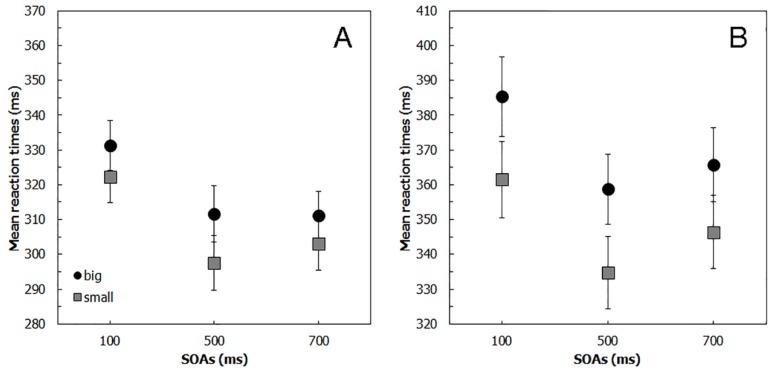
Mean reaction times (RTs) (**A**) for the random block of Experiment 1 and (**B**) for Experiment 2, by Stimulus onset asynchrony (SOA) and Cue conditions. Error bars = standard error of measure (S.E.).

RTs were submitted to a three-way repeated-measures ANOVA with *Block* (random and fixed), *Cue* (small square and big square) and SOA (100 ms, 500 ms and 700 ms) as within-subject factors. A significant main effect of SOA emerged (F(2,38) = 32.60, *p* < 0.001, η^2^ = 0.254), showing that RTs were significantly slower in the 100 ms SOA condition (328 ms) compared to the 500 (302 ms) and the 700 ms SOA conditions (308 ms; *p* < 0.001 for both comparisons), which instead did not differ from each other (*p* = 0.064). Neither the main effect of *Block* (F(1,19) = 0.01, *p* = 0.951, η^2^ = 0.001), and the main effect of *Cue* (F(1,19) = 1.72, *p* = 0.205, η^2^ = 0.006) reached the statistical significance. However, the interaction between *Block* and *Cue* was significant (F(1,19) = 11.44, *p* < 0.005, η^2^ = 0.025), highilighting that, in accordance with previous evidence [8], the cue-size effect (that is the difference between the big and the small square) was significant in the random block (*p* < 0.001; see Figure 2A) but not in the fixed block (*p* = 0.343). The results from the cue-size effect confirmed that the advantage of the small cue was present only in the random block condition. Indeed, in the fixed block condition, the mean cue-size effect was not significantly different from zero in all the three SOA conditions (100 ms: −0.055, t(19) = −0.57, *p* = 0.576; 500 ms: −0.086, t(19) = −0.76, *p* = 0.455; 700 ms: −0.016, t(19) = −0.14, *p* = 0.888). By contrast, even though RTs data demonstrated the presence of a significant advantage for the small square in all the three SOA conditions, the cue-size effect was significantly different from zero only for SOAs of 100 ms (cue-size effect = 0.226, t(19) = 2.96, *p* < 0.01) and of 500 ms (cue-size effect = 0.308, t(19) = 3.25, *p* < 0.005) but not in the 700 ms SOA condition (cue-size effect = 0.176, t(19) = 1.92, *p* = 0.070; see Table 1).

## 3. Experiment 2: Shape Discrimination

In the second experiment, we adapted the original paradigm of Maringelli and Umiltà [8] so that participants had to perform a discrimination task. Indeed, in Experiment 2 we asked the participants to discriminate between two different shapes preceeded by a cue of two possible sizes. The order of the cue presentation was kept random, and, again, three possible SOAs were used.

The aim of Experiment 2 was to investigate whether the magnitude of the size-cue effects can be affected by task demands. In particular, since previous authors suggested that discrimination tasks relay on attention more than detection tasks do [25,26] and, thus, that discrimination tasks are best-suited to revealing attentional effects [5,23,24,27,28], we were expecting to find larger size-cue effects in the case of this discrimination task compared to the detection task of Experiment 1.

### 3.1. Materials and Methods

#### 3.1.1. Participants

Twenty healthy volunteers (13 females, mean age = 23.65 ± 3.20, range = 20–30, all right-handed) took part in Experiment 2. All participants had normal or corrected-to-normal vision and none of them had neurological, psychiatric, or other relevant medical problems.

Participants were all naïve to the experimental procedure and to the purpose of the study, and they gave informed consent prior to being enrolled in the study, which was carried out according to the guidelines of the ethical committee of the University of Milano-Bicocca and in accordance with the ethical standards of the Declaration of Helsinki.

#### 3.1.2. Apparatus, Stimuli and Procedure

The same general procedure of Experiment 1 was chosen for Experiment 2, with the main difference being the task, which consisted of a shape discrimination task, instead of a detection one. Accordingly, the target stimulus could be either a red dot (diameter of 0.4°) or a red rhombus (0.4° × 0.4°). Participants were instructed to press one button if the target stimulus was the red dot or another button if it was the red rhombus. Since Experiment 2 consisted of a discrimination task in which the target stimulus was always present, there were no catch trials during the task.

In Experiment 2, every participant completed 192 trials, equally divided between the 100, 500, and 700 ms SOA conditions, between the two conditions of the cue, and between the two target stimuli (for a total of 16 trials for single condition). The SOA conditions and type of target stimulus were presented randomly. Furthermore, since Experiment 1 showed that the advantage of the small cue was present only in the random block condition, in Experiment 2 the cue types were presented only randomly. At the beginning of the experiment, a practice session composed of 10 trials (equally divided for the cueing condition and randomly selected regarding the SOA condition) was run in order to let the participants familiarize themselves with the task and to practice with response modality.

RTs for Experiment 2 were analysed using the same statistical analyses performed in Experiment 1. Degrees of freedom were corrected using Greenhouse-Geisser correction only when Mauchly’s test revealed a significant violation in the assumption of sphericity. Finally, as for Experiment 1, the cue-size effect was also computed by using the Cohen’s *d* between the big square and the small square conditions for each participant and each SOA condition.

### 3.2. Results

Errors were rare (less than 2%), with no differences between conditions and, thus, were not analysed. RTs were submitted to a two-way repeated–measures ANOVA with *Cue* (small square and big square) and SOA (100 ms, 500 ms and 700 ms) as within-subject factors.

Figure 2B shows the effects of the cues for every SOA condition. In line with Experiment 1, RTs were faster when the target stimulus was preceded by the small cue and in the 500 ms SOA condition. Accordingly, the ANOVA showed that the main effects of *Cue* (F(1,19) = 103.49, *p* < 0.001, η^2^ = 0.208) and of SOA (F(1.27,23.31) = 8.32, *p* < 0.01, η^2^ = 0.202) were significant, whereas the interaction between *Cue* and SOA was not significant (F(2,38) = 0.42, *p* = 0.659, η^2^ = 0.002). RTs to the target stimulus shown within the small cue (348 ms) were faster than when the stimulus was shown within the large cue (370 ms). RTs in the 500 ms SOA condition (347 ms) were significantly faster compared to both the 100 ms (373 ms) and 700 ms SOA conditions (356 ms; *p* = 0.05 and *p* = 0.027 respectively), which, instead, did not differ from each other (*p* = 0.15). The results from the cue-size effect confirmed that the mean cue-size effect was significantly different from zero in all three SOA conditions (100 ms: 0.393, t(19) = 6.72, *p* < 0.001; 500 ms: 0.428, t(19) = 7.56, *p* = < 0.001; 700 ms: 0.272, t(19) = 4.27, *p* = < 0.001; see Table 1).

## 4. Experiment 3: Letter Detection

Whereas in Experiments 1 and 2 we used geometrical shapes as target stimuli because of their wide use in the literature [3,8,11,45], in Experiments 3 and 4, we used letters as target stimuli instead because of their critical role in reading. Indeed, word recognition is mediated by letter detection and identification [38], and previous studies demonstrated that attention can enhance letter identification [53] and play an important role in normal reading [46,47,54] and developmental dyslexia [48,55,56]. For this reason, the aim of Experiments 3 and 4 was to better characterize the effect of the focal component on letter detection and identification in foveal vision.

In particular, in Experiments 3 and 4 we added a SOA of 300 ms in order to have a more complete picture of the time course of the attentional effect on letter detection and identification. Moreover, a condition of absence of the cue was also included in order to disentangle the role of the alertness due to the presence of a cue, independently from its shape and size, from the specific effect of the focal component.

### 4.1. Materials and Methods

#### 4.1.1. Participants

Twenty healthy volunteers (13 females, mean age = 23.52 ± 3.46, range = 20–31, all right-handed) took part in Experiment 3. All participants had normal or corrected-to-normal vision, and none of them had neurological, psychiatric, or other relevant medical problems.

Participants were all naïve to the experimental procedure and to the purpose of the study. They gave informed consent prior to being enrolled in the study, which was carried out according to the guidelines of the ethical committee of the University of Milano-Bicocca and in accordance with the ethical standards of the Declaration of Helsinki.

#### 4.1.2. Apparatus, Stimuli and Procedure

The same general procedure from Experiment 1 and Experiment 2 was used for Experiment 3.

In Experiment 3, a baseline condition, consisting of the absence of any cue and an additional SOA level (300 ms) was added. The target stimulus was the letter ‘r’ (font: Courier; letter size: approximately 0.75° × 0.75°), and it was always shown in the centre of the cue. The response to the target stimulus was made by pressing the space-bar on the computer keyboard. Similarly to Experiment 1, on 20% of trials (catch trials) no target stimulus was presented.

In Experiment 3, every participant completed 288 trials, equally divided between the 100, 300, 500, and 700 ms SOA conditions and between the three cueing conditions (big square, small square, and absence of cue; for a total of 24 trials for single condition). SOA conditions and cue types were presented randomly. At the beginning of the experiment, a practice session composed of 12 trials (equally divided for the cueing condition and randomly selected regarding the SOA condition) was run in order to let the participants familiarize themselves with the task and to practice with response modality.

The same statistical analyses from Experiments 1 and 2 were performed also on data from Experiment 3, with the exception that this time the SOA factor included four levels (i.e., 100, 300, 500, and 700 ms).

### 4.2. Results

**Figure 3:**
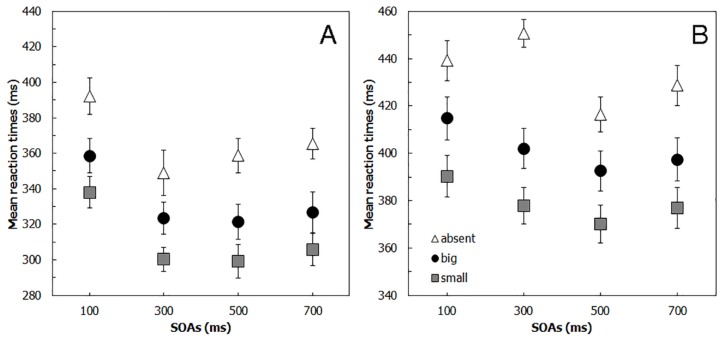
Mean reaction times (RTs) for (**A**) Experiment 3 and (**B**) Experiment 4, by Stimulus onset asynchrony (SOA) and Cue conditions. Error bars = standard error of measure (S.E.).

Participants committed very few false alarms in the catch trials (less than 3%), with no differences between conditions, and, again, these data were not further analysed. RTs were submitted to a two-way repeated–measures ANOVA with *Cue* (small square, big square, and absence of cue) and SOA (100 ms, 300 ms, 500 ms, and 700 ms) as within-subject factors. The effects of the different cues on the mean RTs are shown in Figure 3A.

The ANOVA revealed a significant main effect of *Cue* (F(2,38) = 48.13, *p* < 0.001, η^2^ = 0.387) and of SOA (F(3,57) = 27.18, *p* < 0.001, η^2^ = 0.178). RTs when the SOA was 100 ms (363 ms) were significantly slower than when the SOA was 300 (325 ms), 500 ms (326 ms), or 700 ms (333 ms; all *p* < 0.01). RTs to the target stimulus shown within the big cue (333 ms) and within the small cue (311 ms) were significantly different from each other and from the absence of a cue (366 ms; all *p* < 0.001). The interaction between *Cue* and *SOA* was not significant in Experiment 3 (F(3.27,62.11) = 0.47, *p* = 0.719, η^2^ = 0.004), showing that the benefit due to the presence of the cues was present with all SOAs. Accordingly, the mean cue-size effect was significantly different from zero in all the possible SOA conditions (100 ms: 0.386, t(19) = 5.43, *p* < 0.001; 300 ms: 0.405, t(19) = 4.07, *p* < 0.001; 500 ms: 0.482, t(19) = 4.52, *p* = < 0.001; 700 ms: 0.328, t(19) = 4.86, *p* = < 0.001; see Table 1).

## 5. Experiment 4: Letter Discrimination

Since the results from Experiment 3 demonstrated that letter detection can be enhanced when attention is focused on the target and because previous studies showed that letter discrimination and identification can be improved by orienting attention in the periphery of the visual field [53], the aim of Experiment 4 was to investigate whether focal attention can also improve letter discrimination. In particular, given that in Experiment 2 the discrimination task induced a bigger cue-size effect compared to the detection task of Experiment 1 and that in Experiment 3 letter detection induced a bigger cue-size effect than with the shape detection of Experiment 1, we expected that letter discrimination would show an even bigger cue-size effect compared to shape discrimination or letter detection.

### 5.1. Materials and Methods

#### 5.1.1. Participants

Twenty healthy volunteers (14 females, mean age = 24.20 ± 2.98, range = 19–29; all right-handed) participated in Experiment 4. All participants had normal or corrected-to-normal vision, and none of them had neurological, psychiatric, or other relevant medical problems.

Participants were all naïve to the experimental procedure and to the purpose of the study. They gave informed consent prior to being enrolled in the study, which was carried out according to the guidelines of the ethical committee of the University of Milano-Bicocca and in accordance with the ethical standards of the Declaration of Helsinki.

#### 5.1.2. Apparatus, Stimuli and Procedure

The same general procedure of Experiment 3 was used, with the only exception that this time the target stimulus could be either the letter ‘r’ (font: Courier; letter size: approximately 0.75° × 0.75°) or the letter ‘t’ (font: Courier; letter size: approximately 0.75° × 0.75°). Participants were instructed to fixate on the centre of the screen, to focus attention inside the square, and to press one button for the ‘r’ target stimulus or another for the ‘t’. Since Experiment 4 consisted of a discrimination task in which a target stimulus had to be always present, no catch trials were included in this task.

Every participant completed 288 trials; SOA conditions and type of cue were presented randomly (for a total of 24 trials for single condition). At the beginning of the experiment, a practice session composed of 12 trials (equally divided for the cueing condition and randomly selected regarding the SOA condition) was run in order to let the participants familiarize themselves with the task and to practice with response modality.

Data from Experiment 4 were analysed by using the same procedure as in Experiment 3.

### 5.2. Results

Errors were rare (less than 3%) and, thus, were not further analysed. RTs were submitted to two-way repeated–measures ANOVA with *Cue* (small square, big square and absence of cue) and SOA (100 ms, 300 ms, 500 ms and 700 ms) as within-subject factors.

The ANOVA showed that the main effect of *Cue* (F(1.26,24.03) = 60.78, *p* < 0.001, η^2^ = 0.366) and the main effect of SOA (F(3,57) = 3.52, *p* < 0.05, η^2^ = 0.051) were significant. RTs to the target stimulus shown within the small cue (379 ms) were faster than when the stimulus was shown within the large cue (402 ms) and when the stimulus was shown without any cue (434 ms; all *p* < 0.001). Participants were also faster in responding after the large cue compared to the absence of a cue (*p* < 0.001). RTs when the SOA was 100 ms (415 ms) were slower than the other SOA conditions (SOA 300 ms: 410 ms; SOA 500 ms: 393 ms; SOA 700 ms: 401 ms), but in Experiment 4 only the difference with the 500 ms SOA condition was significant (*p* < 0.005). Again, the interaction between *SOA* and *Cue* (F(3.79,71.97) = 1.87, *p* = 0.129, η^2^ = 0.017) was not significant, showing that that the benefit due to the presence of the cues was present with all SOAs (see Figure 3B).

Results from the cue-size effect showed that the mean cue-size effect was significantly different from zero in all the possible *SOA* conditions (100 ms: 0.388, t(19) = 6.49, *p* < 0.001; 300 ms: 0.307, t(19) = 7.26, *p* < 0.001: 500 ms: 0.340, t(19) = 6.74, *p* = < 0.001; 700 ms: 0.266, t(19) = 4.15, *p* = < 0.001; see Table 1).

## 6. Comparing Experiments 1, 2, 3 and 4

We finally compared the cue-size effects found in the four experiments in order to highlight the presence of any possible effects of the task demand or of the stimulus used and to further investigate the temporal trends of the focal components (i.e., how the magnitude of the cue-size effect was affected by different SOAs). To this aim, Cohen’s d values were submitted to a three-way repeated–measures ANOVA with SOA (100 ms, 500 ms, and 700 ms) as a within-subject factor and *Task* (detection and discrimination) and *Stimulus* (shape vs letter) as between-subject factors.

**Figure 4:**
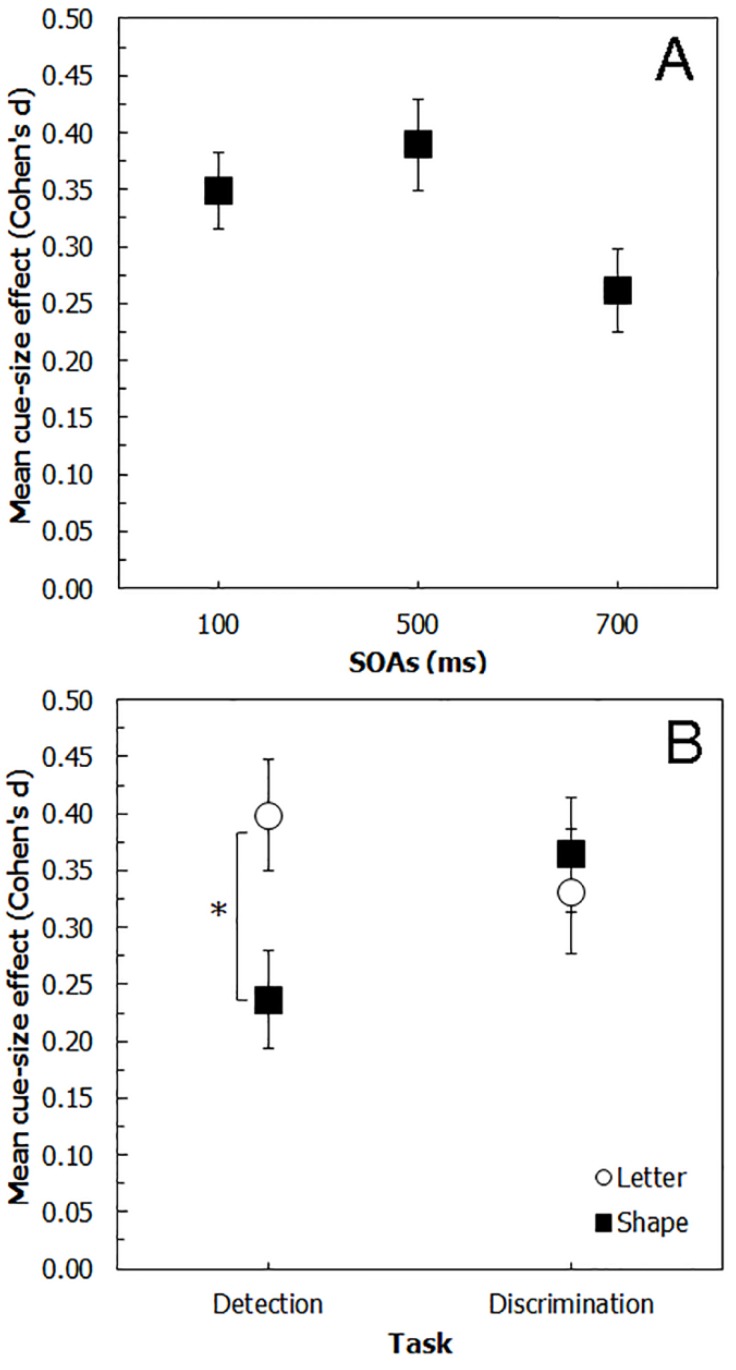
Cue-size effects by (**A**) SOA and (**B**) Task per Stimulus. Error bars = standard error of measure (S.E.).

This analysis revealed that neither the main effect of *Stimulus* (F(1,76) = 2.44, *p* = 0.123, η^2^ = 0.029) nor the main effect of *Task* (F(1,76) = 53, *p* = 0.844, η^2^ = 0.001) were significant . By contrast, the main effect of SOA (F(2,152) = 3.10, *p* < 0.05, η^2^ = 0.039) and the interaction between the *Stimulus* and the *Task* factors (F(1,76) = 5.52, *p* < 0.05, η^2^ = 0.065; see Figure 4) were significant. The main effect of SOA revealed that the cue-size effect at 700 ms (0.261) was reduced compared to the other SOA conditions (SOA 100 ms: 0.348; SOA 500 ms: 0.389), but only the difference with the 500 ms SOA condition was significant (*p* < 0.05). The interaction between *Stimulus* and *Task*, instead, showed that, in the detection task, the use of letter stimuli elicited larger cue-size effects compared to shape stimuli (0.399 and 0.237, respectively; *p* < 0.01), whereas the difference between letters and geometrical shapes in the discrimination task was not significant (0.332 and 0.364, respectively; *p* = 0.579). Accordingly, for the geometrical shapes the difference between the detection and discrimination tasks was significant (0.237 and 0.364, respectively; *p* < 0.05), whereas, for the letter stimuli, it was not (0.399 and 0.332, respectively; *p* = 0.256).

## 7. Discussion

Whereas a lot of evidence has been collected in the past to understand how to orient spatial attention [57,58,59,60], less is known about how attentional resources can be selectively concentrated on a limited ‘spatial window’, i.e., focal attention. In particular, we still do not know so much about the many factors that might affect the deployment of focal attention. For this reason, the specific aim of this study was to assess the effects of the task demand and of the type of stimulus on focal attention (i.e., the magnitude of the cue-size effect) and on its temporal trend. The results of these four experiments highlighted that focal attention strongly affects participants’ performance in foveal vision conditions, and thereby it shortens their reaction times. In particular, as compared with previous research on this component [3,8,45], the main novel finding is that, even though the cue-size effect can be elicited by using different tasks (i.e., detection or discrimination) and different target stimuli (i.e., shapes or letters), the choice of the task and of the stimulus used can affect the magnitude of the cue-size effect linked to focal attention.

In particular, the results of Experiment 1 (shape detection) suggested that the advantage linked to the deployment of focal attention was more evident with short SOAs, namely 100 ms and 500 ms. These data are in accordance with a previous study of Maringelli and Umiltà [8], which found that the inverse relationship between the size of the attentional focus and the efficiency of processing is more evident for short SOAs. Taken together, the findings of both studies converge in showing that the cue-size effect is enhanced at short SOAs, suggesting that focusing attention is mainly a reflexive or exogenous mechanism; however, this does not imply a complete independence of focal attention from an endogenous or top-down control. Indeed, in both studies, the advantage of the small cue disappeared when the sequence of presentation of the cue was fixed. Furthermore, a cue-size effect was detectable also for the longest SOA (700 ms), even if smaller compared to the shorter SOA. Indeed, in Experiment 2 (shape discrimination) the benefit associated with the presence of the small cue was present also at the longest SOA, strengthening the idea that even if stronger in exogenous conditions, focal attention can also be deployed endogenously in foveal vision conditions.

The results from Experiment 3 and Experiment 4 showed that the temporal advantage associated with focal attention is also evident when letter stimuli are used, independently of the SOA between the cue and the appearance of the target stimulus. Moreover, Experiments 3 and 4 proved that a temporal advantage was also present when the target stimulus was preceded by the big cue compared to when the target stimulus was not cued. However, since the big cue was too large in relation to the target size, thus eliciting focus over a larger area compared to the target stimulus, the effect associated with the big square was considerably smaller than the one associated with the presence of the small cue, and it was not detectable in all SOA conditions. Furthermore, it is possible that, more than reflecting only the influence of focal attention, this result might be also partially explained by the fact that the big cue could have acted as temporal cue on the future appearance of the target stimulus, serving as a general alertness for future events in the trials. In this case, however, this temporal advantage was considerably smaller than the one associated with the presence of the small cue because the SOA varied randomly during each experiment and, thus, the temporal cue was not accurate like the spatial cue (i.e., the small square). Similar results were also shown in previous studies [4,61,62] and confirm that, whereas the advantage linked to the presence of the small square truly reflects the deployment of focal attention, the advantage linked to the presence of the big square simply indicates an improved performance for events appearing at expected timings.

A direct comparison of the cue-size effects across the four experiments showed that neither the task demands nor the stimulus type alone affected the effect of the focal component substantially. However, an interaction between these two factors was found, highlighting that probably it is not the task or the stimuls type per se that induces the strongest cue-size effect. In particular, in the case of a detection task, letter stimuli elicited larger cue-size effects compared to geometrical shapes, whereas, in a discrimination task, the two types of stimuli elicited similar cue-size effects. Accordingly, only the cue-size effects elicited by the geometrical shapes differed significantly between the detection and discrimination tasks, whereas the ones evoked by letters did not. Previous studies on focal attention have already tried to use both detection and discrimination tasks, but a direct comparison of the cue-size effects obtained with the two paradigms and with the use of different stimuli was missing. In particular, whereas those studies [3,4] demonstrated that the cue-size effect can be obtained with both tasks, here we show that target type modulates the effect of task demand on focal attention. These results may explain the different findings in the literature, with some authors claiming that a detection task should be used [7,11,29,30] and others that the use of a discrimination task would be more suitable [5,23,24,27,28] and with some studies suggesting that the attentional effects depend on the stimuli used [18,19,20]. In particular, our results show that, overall, both detection and discrimination tasks can efficiently reveal the deployment of focal attention; nevertheless, the stimuli to be used should be selected carefully in accordance with the task. Indeed, whereas in the case of a discrimination task the use of one type of stimulus over another does not seem to affect the cue-size effect, the same is not true for detection tasks. The fact that letter stimuli evoked a similar attentional effect in detection and discrimination tasks seems to mimic previous findings showing that some stimuli (such as letters and numbers) may undergo ‘compulsive encoding’, meaning that they are automatically processed regardless of task demands [39]. On the other side, the larger cue-size effects elicited by letters compared to geometrical shapes is in accordance with previous findings showing that the strength of the deployment of focal attention could be altered by target type and available cognitive resources [63].

Detecting a target stimulus requires the visual system to solve a simpler problem than discriminating the same target stimulus, and, for this reason, detection tasks do not require as much attentional resources like discrimination tasks do [5,23,24,27,28]. It is possible that, under such conditions, some stimuli (letters in our case) are more sensitive to attentional effects compared to others (geometrical shapes in our case) and that this discrepancy might be due to the fact that letters are more relevant stimuli for us. Indeed, word recognition is mediated by letter detection and identification [38]; thus letters may represent stimuli with higher-level representations compared to geometrical shapes. This interpretation seems consistent with a previous study showing that high-level objects take precedence over low-level objects in guiding focal attention [14], which is similar to what happens in the orienting domain [33,34,35,36,64]. By contrast, in the case of a discrimination task, the difference between letter stimuli and geometrical shapes would not be evident because of the greater attentional demand posed by discrimination [5,23,24,27,28]. Thus, with discrimination tasks the difference between letters and geometrical shapes stimuli would not be evident because attentional resources are already recruited to complete the task.

It could be argued that a small cue, compared to a large cue, evokes shorter RTs merely because positional uncertainty is reduced in the former case. Indeed, according to some authors [65,66], the enhanced performance under cued conditions can be interpreted as the result of reduced position uncertainty, rather than as evidence of a pure property of attention. However, since in our study the target stimulus was fixed in the centre of the cue, positional uncertainty was minimal (or absent); thus this factor cannot account for our results. Moreover, spatial uncertainty affects the performance differently, according to the task demand [57]. Indeed, in the case of a discrimination task the cue can only indicate the probable target location, without conveying any information about the correct response (e.g., the identity of the stimulus), whereas in the case of a detection task, cues that predict the target location with high probability lead the participant to direct their attention to that particular location, favouring the possibility that the enhanced detection is due to a decision mechanism employed by observers [67]. Here, both a detection and a discrimination task were used, and we were able to observe the similar effect of focal attention in both, further confirming that our results were not caused by position uncertainty. Thus, it can be assumed that the cue-size effect we found represents a true attentional effect.

Finally, the analysis of the cue-size effect across the four experiments showed that it was visible in all SOA conditions. Previous studies have already investigated the temporal characteristics of focal attention, leading however to different results. For example, by using an adapted version of the Posner’s paradigm with cues of different sizes,Castiello and Umiltà [7],Castiello and Umilta [68] showed that, when the interval between the cue and the target stimulus was too short (40–50 ms), the expected inverse relation between the size of the attentional focus and the efficiency of processing disappeared; by contrast, other studies [5,8] found that in foveal vision focusing can also occur with a short SOA of 100 ms. Similarly, other studies [3,11] showed that the focal component requires between 33 and 66 ms to fit the shape of the cue, that it can be maintained until about 500 ms, and that it fades out at about 700 ms. Our results seem to confirm that the cue-size effect can be elicited in a reflexive way for short SOA and in a voluntary and endogenous one for longer intervals; particularly, the first would be automatically fit to a new object appearing in the visual field, while the second would maintain attention in the focused mode [3]. Furthermore, by confirming previous results [4,8], our data suggest that the deployment of focal attention in foveal vision is best revealed and more effective under exogenous conditions. However, since a limited range of SOAs was used (especially on the lower side), further studies will be necessary in order to obtain a better understanding of the temporal characteristics of the cue-size effect.

In conclusion, in the present study we demonstrated that neither the task demands nor the stimuli used affect the magnitude of the cue-size effect per se. However, those two factors interplay in guiding focal attention; for example, when the attentional demands posed by the task are limited, high-level objects elicit a larger cue-size effect. By contrast, when more attentional resources are already recruited for the completion of the task, there are no differences between low- and high-level objects.

## Figures and Tables

**Table 1:** Cue-size effects (mean ± SE) by stimulus onset asynchrony (SOA) in the four experiment.

SOAs (ms)				
Experiment	*100*	*300*	*500*	*700*
*Experiment 1 (random block)*	0.226 ± 0.076	---	0.308 ± 0.095	0.176 ± 0.092
*Experiment 1 (fixed block)*	−0.055 ± 0.097	---	−0.086 ± 0.113	−0.016 ± 0.110
*Experiment 2*	0.393 ± 0.058	---	0.427 ± 0.057	0.272 ± 0.064
*Experiment 3*	0.386 ± 0.071	0.405 ± 0.099	0.482 ± 0.107	0.328 ± 0.068
*Experiment 4*	0.388 ± 0.059	0.307 ± 0.042	0.340 ± 0.050	0.266 ± 0.064

---, not tested.
